# Rapid Detection of Chicken Residues on Poultry Plant Surfaces Using Color and Fluorescence Spectrometry

**DOI:** 10.3390/foods14244352

**Published:** 2025-12-18

**Authors:** Clark Griscom, Dongyi Wang, Corliss A. O’Bryan, Rimmo Rõõm, Philip G. Crandall

**Affiliations:** 1 Department of Food Science, University of Arkansas, 2650 N. Young Ave., Fayetteville, AR 72704, USA; crgrisco@uark.edu (C.G.); dongyiw@uark.edu (D.W.); cobryan@uark.edu (C.A.O.); 2 Biological and Agricultural Engineering, University of Arkansas, 790 W. Dickson St., Fayetteville, AR 72704, USA; 3 LDI Innovation OÜ, Sära tee 7, 75312 Peetri, Harjumaa, Estonia; rr@ldi-innovation.com

**Keywords:** fluorescence, color spectrometry, food-contacting surface, poultry processing, chicken contamination, cleaning verification, food safety

## Abstract

Color and fluorescence spectrometry were evaluated as rapid, objective tools for verifying the cleanliness of poultry-processing food-contacting surfaces contaminated with a model chicken solution across six common materials. Both techniques detected chicken residues at dilutions several orders of magnitude below human visual and olfactory thresholds, with stainless steel and blue plastic yielding the largest color differences between clean and contaminated states and fluorescence measurements remaining highly sensitive on all tested surfaces. Representative limits of detection were on the order of 1:50–1:100 dilution of chicken residue for color measurements on most surfaces and approximately 1:50 for fluorescence measurements, compared with human detection thresholds of approximately 1:50. Cleaning chemicals routinely used in poultry plants did not measurably reduce detection performance, and a simple machine learning classifier further improved separation of clean versus contaminated readings. These findings indicate that compact color and fluorescence instruments can provide fast, quantitative pre-sanitation checks that strengthen SSOP verification and reduce reliance on subjective human inspection in poultry processing facilities.

## 1. Introduction

Ensuring the safety of food products is one of the most important tasks of every food processing facility. To ensure the safety of the food supply, each food processing establishment must develop, implement, and maintain written standard operating procedures for sanitation [1]. Sanitation Standard Operating Procedures (SSOPs) are written procedures designed to prevent contamination and adulteration of food products. After SSOP inspections are conducted, the plant manager signs a statement attesting that each SSOP was completed and that all food-contacting surfaces of the facility are clean to sight and touch before resuming operations [2]. The typical order of cleaning and sanitizing begins with the dry removal of visible soil, followed by a cold-water rinse, foam-soap cleaning, a specified holding period, a water rinse, and sanitizing all food-contacting surfaces [3]. Plant Inspection Program Personnel (IPP) perform validations by conducting hands-on inspections, visually inspecting the food-contacting surfaces, feeling to determine whether soil is below the visible level, and smelling to verify that the food-contacting surfaces are clean.

Despite these strict protocols, foodborne illnesses remain a significant public health concern. An estimated 9.9 million foodborne illnesses were caused by *Campylobacter* spp., *L*. *monocytogenes*, norovirus, nontyphoidal *Salmonella,* and Shiga toxin-producing *E. coli* in the United States [4]. A primary attribution model based on the inter-agency Food Safety Analytics Collaboration assumptions estimated that 30.9% of the vehicles implicated in foodborne illnesses were from meat and poultry products [5]. Meat and poultry-related foodborne illnesses cost an estimated USD 20.3 billion in economic losses [5].

One way to reduce foodborne illnesses is to improve verification of the plant’s cleaning SSOPs. The current verification of cleaning efficacy relies on inspection personnel’s ability to use their senses of sight, smell, and touch to determine whether a surface is clean in accordance with SSOPs. Poultry processing facilities are typically cold, wet, and have numerous odors, and many areas are poorly lit, all of which can impede human sensory abilities [6]. Currently verification of SSOPs relies only on the quality technicians’ sight, touch, and smell, and nonspecific ATP bioluminescence swabbing. There is previous research that uses fluorescent technology, but it is still not used in the industry due to the need to add non-food-safe dyes and its use as an imaging system. Our method uses quantitative results at multiple wavelengths to determine if specific contamination from chicken is present. No unsafe food components are used with this technology, allowing for it to be used in processing plants as well.

Research is needed to improve bacterial detection and detect other contaminants. The bacterial form that should be targeted to improve food safety is biofilms due to their high resistance to antimicrobials [7]. *Salmonella* are biofilm-forming bacteria typically isolated from poultry processing plants [8].

Following water rinsing after soap application, many plants use chemical sanitizers, but these sanitizers can be rendered ineffective by soil deposits. Other sanitizers react with residual organic matter and cannot effectively destroy microorganisms, making it necessary to clean surfaces free of visible soil [7]. This means that to increase the efficacy of sanitizers, it is imperative to apply them to clean surfaces that are free of food soil [7]. Sanitizing is defined as the reduction in microorganisms to a level considered safe, i.e., a 5-log reduction [8]. If the sanitizing process works correctly, there should be fewer outbreaks of foodborne illness.

Two promising technologies offer potential improvements to this challenge. Color spectrometry can be used to measure the visible color change between the typical food-contacting surface and food particles on it. A color spectrophotometer quantifies color changes across the human visual spectrum (400–700 nm) and offers superior precision and documentation capabilities. Additionally, fluorescence technology offers another method for verifying cleanliness. Fluorescence—the emission of short-wavelength light upon absorption, causing excitation and then photoemission—has been used in meat processing with UV and blue light [9,10]. By leveraging these specific wavelengths, fluorescence can detect residual chicken soil particles on food-contacting surfaces. Fluorescence and imaging inspection techniques have also been used to detect fecal contamination on poultry carcasses [10]. In a research study, fluorescence was used to detect dried fruit juice on food-soiled stainless steel surfaces of processing equipment [11].

The objectives of this study were to develop a more accurate and reliable method for determining surface cleanliness before applying sanitizers in poultry processing plants. Using both a color spectrophotometer and a bio-fluorometer, the limit of detection (LOD) was determined for the instruments’ detection of food soil using a model chicken solution. This test was conducted in parallel with sensory evaluations to compare the accuracy and precision of the instruments with human perceptions. This research seeks to introduce an objective approach to verifying and documenting cleanliness before resuming processing. Fluorescence detection was combined with machine learning to help address the need for surface specific results. The learning model used helped distinguish the results of the background fluorescence on each surface. Chicken contamination fluorescence was used by the learning model to distinguish when the specific tested surface is clean or dirty by using PCA. This study aimed to assess color and fluorescent spectrometry with exploratory support of a machine learning model to reliably detect low levels of chicken contamination on food-contacting surfaces to improve the standard cleaning procedures in poultry processing plants.

## 2. Materials and Methods

### 2.1. Food-Contacting Surfaces

Six food-contacting surfaces typically found in poultry processing plants were used as model food-contacting surfaces. The six surfaces being tested were D&F Milled Finished Stainless Steel (stainless steel D&F, 450 Agnes Drive, Tontitown, AR 72770, USA), a white ultra-high-molecular-weight polyethylene surface (polyethylene), Intralox Thermo Drive Series 8050 Flat Top E Blue Polyurethane (blue belt Intralox 301 Plantation Road, New Orleans, LA 70123, USA), Intralox Thermo Drive Series 8050 Flat Top E White Polyurethane (white belt), Intralox Series 800 Open Hinge Flat Top with Heavy Duty Edge Pk Blue (blue-hinge belt), and Series 800 Open Hinge Flat Top with Heavy Duty Edge Pk White (white-hinged belt) (Intralox, L.L.C. 7157 Ridge Rd, Hanover, MD 21076, USA). 

Before use, each surface was cleaned using water and sanitized with disinfecting wipes. The surfaces were allowed to air dry before testing.

Each food-contacting surface was tested in three conditions: clean and dry, clean with tap water, and inoculated. Before applying the model soil, ten readings were taken at each of five locations on the clean, dry surfaces being tested. Four readings were taken on the clean, dry surface, and three at each clean with tap water and inoculated. The average was then calculated from each set of condition readings, and dE* was calculated based on the reference sample. After taking the readings for each cleaned surface, 1 mL of water was added at three locations on each surface. Using an L glass spreader and Tween 20 (Croda International LLC, New Castle, DE, USA) to spread the water to fill a 10 × 10 cm plastic square template to accurately match the current swabbing area in food processing plants. Between testing each dilution, the surface was rinsed with water, sanitized, and returned to a clean, dry state. Once all measurements were completed, the data collected was transferred from the spectrophotometer to an Excel file.

### 2.2. Preparation of Chicken Dilutions

Raw bone-in chicken thighs were purchased from a local grocery store. All packages had the same sell-by date, but whether they were from the same batch is unknown. No analyses for fat and protein were performed. The thigh meat and skin were frozen, partially thawed, and cut into 5 mm pieces, blended (Ninja Professional Blender 1000 W 72-oz. BL610, Needham, MA, USA) in a ratio of 3 parts thigh meat to 1 part skin, together with known amounts of tap water. The samples were then examined under a microscope to confirm adequate homogenization. The dilution ratios from least diluted to most are 1:10, 1:25, 1:40, and 1:50 parts chicken to water. Testing was conducted using tap water and a clean, dry surface as baseline measurements.

Due to real residues having no clear quantitative amount for a limit of detection test, we created a model chicken contamination solution. The model chicken contamination solution not only allowed the authors to obtain a better understanding of what the human limit of detection is, but also includes a true quantitative amount of known chicken contamination present. Real residues, including chicken blood, fat, and meat, were used to create the model solution to simulate the true contaminating residues in processing plants. Real contamination has yet to include a quantitative result, causing potential errors in unknown concentrations of how much residue is present.

### 2.3. Color Spectrometer

Color was measured using a Hunter Lab portable handheld spectrophotometer (Mini Scan EZ 4000L: Portable Spectrophotometer, Hunter Associates Laboratory, Inc., Reston, VA, USA). Each test was reproduced by using the same test setup: placing the instrument directly on the surface, with no outside light entering and no light escaping the flash. This allowed for each sample to be tested consistently by placing it directly in the center of the 10 × 10 cm square. Following a CIE-L*C*h system, data were measured based on the total color change (delta E variance, dE*), where a higher dE* indicated a greater color shift. A dE* value of 5 may be noticeable to the eye, and a dE* value of 1 would require very good lighting and close inspection to differentiate a clean surface from a soiled one. The instrument was standardized using the standard white and black tiles supplied with the instrument. Three readings of the cleaned and dried surface were averaged to obtain the standard surface color. The reference surface standard was tested on the clean surface at multiple locations to obtain the standardized color. Specifically, on the hinge belts, an interlocking system creates a crack that alters the color. The standardized readings accounted for the belt’s hinges to create the reference surface, whereas some of the clean readings did not.

**Figure 1:**
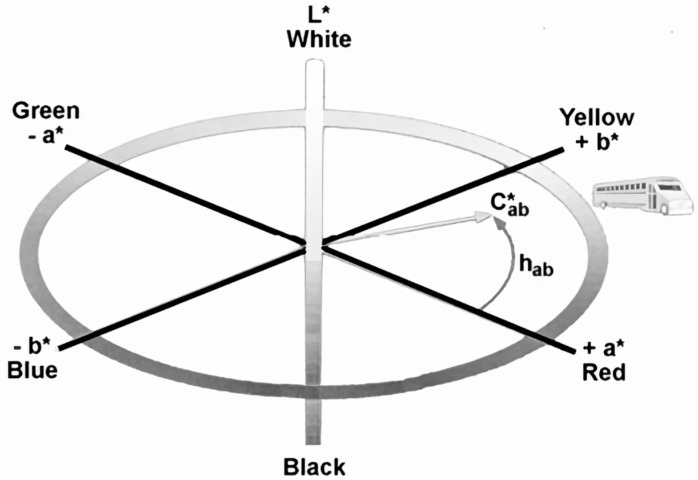
Demonstrates the CIE-L*C*h scale of using lightness (L*), chroma (C*), and hue angle (h) to determine the dE*. Reprinted with permission from HunterLab. Copyright year 2008, copyright owner HunterLab [12].

dE* is the most distinguishing factor for determining the total color difference due to the calculation of all three parameters of L*, a*, and b*. The instrument has multiple color scales, including L*a*b* and L*C*h. From preliminary testing, a* and b* showed less significant results than C* and h, because C* and h are the calculated color intensity and the pure color itself. When selecting a new color scale, such as L*C*h, the instrument does not provide the numerical values of a* and b* in the data itself; rather, it only includes them in the calculations used to collect the data. The calculation for dE* also uses a* and b*, even though the values of a* and b* were not given in the raw data collected. Thus, L*a*b* are used in the instrument to calculate C*h and dE* (Figure 1).

### 2.4. Bio-Fluorometer

LDI Innovation provided the H2B Spectral, a handheld bio-fluorometer used to measure fluorescent materials (“Bio-cleanness sensor” H2B-Spectral, LDI Innovation, Sara tee 7, 75312 Peetri, Harjumaa, Estonia) [13]. To measure the fluorescence of materials, the bio-fluorometer has three spectral optical channels. Three LEDs (light-emitting diodes) illuminate the object, exciting the light-induced fluorescence of the material. The bio-fluorometer measures the intensity of fluorescence based on eight channels of varying excitation and emission wavelengths, resulting from a combination of three LEDs and high-quality filters. Following preliminary experiments, the excitation wavelength channels 280 nm, 310 nm, and 340 nm were selected. The emission receptor wavelength channels were 340 nm, 405 nm, and 460 nm. Each channel was labeled by either emission-excitation wavelength or a letter from A through H. Preliminary experiments using the multifunctional wavelength analyzer and visual molecular dynamics software predicted the maximum emission wavelength of the fluorescence spectra for poultry soil to be 341 nm, likely due to the amino acid tryptophan [14]. (Koh & Yu) [15], used mg% to determine the parts of tryptophan present in chicken thighs. The mg% is the amount of milligrams in 100 g of chicken meat. So, 205 mg% tryptophan is 205 mg in 100 g of chicken meat.

Tryptophan has a composition of 205 mg% in chicken thighs and 260 mg% in breast meat [16]. Based on this information, channel F was the best choice, since it has an emission-excitation wavelength of 340 nm–280 nm and will pick up the fluorescence of tryptophan.

### 2.5. Sanitizing Compounds

The sanitizing compounds peroxyacetic acid (PAA) and quaternary ammonium compounds (QUAT) were tested as a potential interfering residues from the cleaning process. Each compound was diluted according to the instructions for cleaning food-contacting surfaces. The sanitizing compound was tested using the same procedure as chicken and water, but only on the stainless steel and polyethylene surfaces. We followed the same steps as the color spectrophotometer to test each surface, water, and dilution, adding sanitizing compounds.

### 2.6. Support Vector Machine

The support vector machine classifier was selected for this study based on its previously demonstrated ability to accurately differentiate classes of microorganisms [17]. In this study, SVM was applied to classify clean and intentionally soiled surfaces as an exploratory preliminary study. A principal component analysis (PCA) was used to visually confirm the separation of clean and soiled surfaces, confirming distinct clustering. Hyperparameter optimization was performed using GridSearchCV with repeated k-fold cross-validation, with the tuning parameters including regularization (C), gamma, and the degree and coefficient (coef 0) of the polynomial kernel. The optimized model was subsequently integrated into the H2B-Spectral software. Model training and evaluation, including preprocessing and visualization, were conducted in Python 3.12.11 (Google Colaboratory) using Pandas 2.2.2, NumPy 2.0.2, Scikit-learn 1.6.1, Plotly 5.24.1.

The support vector model (SVM) and principal component analysis (PCA) were only used in the software of the bio-fluorometer. This software was used to calculate the probability that the surface is clean or dirty, but the preliminary results were not included in the data collection. Further research is necessary to assess the effectiveness of the built-in learning models.

The data collected were split using principal component analysis of the defined contaminated and cleaned results of all eight excitation and emission channels. The training involved the principal component analysis, breaking each data set into groups of surface type tested, and determining if contamination was present or not. A matrix was then performed to determine how many of the predicted data points matched the actual results, which were used to train the model. After training, data were collected to test the model and assess its classification accuracy.

### 2.7. Human Sensory

Before collecting data, each sample was visually inspected using the authors’ human sensory abilities of sight, touch, and smell. Based on the author’s abilities, a dilution of 1:50 was determined to be above the upper limit of detection for humans on each of the food-contacting surfaces tested. Based on this information, the color spectrometer and bio-fluorometer results used the 1:50 dilution above the threshold of human sensory abilities. The study was verified as exempt by the University of Arkansas Human Subjects Institutional Review Board (Approval No. 2406545996).

### 2.8. Poultry Pilot Plant

Ten locations on a polyurethane food-grade contact belt used in a poultry processing plant were used to test the bio-fluorometer in the University’s Poultry Processing Pilot plant. Each location was tested as clean and dry, then cleaned with tap water, and subsequently contaminated with a 1:50 dilution of chicken. To measure fluorescence intensities, the bio-fluorometer was placed directly on the location, and ten measurements were taken. Data collected was transferred directly from the bio-fluorometer to a CSV file.

### 2.9. Statistical Analysis

Color measurement means of clean, wet, and contaminated surfaces using CIE-L*C*h scale along with dE* were compared using pairwise Tukey’s honest significant difference (HSD) tests with significance level of *p* < 0.05. Mean fluorescent intensities were calculated for clean, wet, chicken-contaminated, and sanitized surfaces. Means were compared using pairwise Tukey’s HSD test with a significance level of *p* < 0.05. All statistical analysis was completed in R (Version 4.4.2, R Core Team, 2024) [18].

## 3. Results

### 3.1. Color Spectrometry

The color spectrometer results of the 1:50 dilution can be compared to a human’s visual interpretations, as the instrument’s color scale is like a human’s visual spectral range. Each surface was measured in one of three states: clean, tap water, or chicken. Clean means that the surface is clean, sanitized, and dry. Water refers to tap water being added to the cleaned surface and measured. Chicken refers to adding the model chicken soil dilution to a previously clean and dry surface. Each surface was tested at three locations in each state, and the average of ten readings was calculated. The total color difference had the most significant results. On the blue belt and blue-hinge belt the water created the largest difference due to the color intensity being greater. The results also show the intensity of the color being greater in the chroma, which is the saturation of a pure color. When chicken contamination was present on the surface, the saturation of the color decreased, resulting in a smaller total color difference. The surfaces with white background colors had the least effect of water and chicken contamination. Due to the color of the fat particles being very light, the chicken contamination had a similar color to the background of the white surface. The stainless steel surface had the greatest total color difference when chicken contamination was present, averaging a dE* of 2.6.

**Figure 2:**
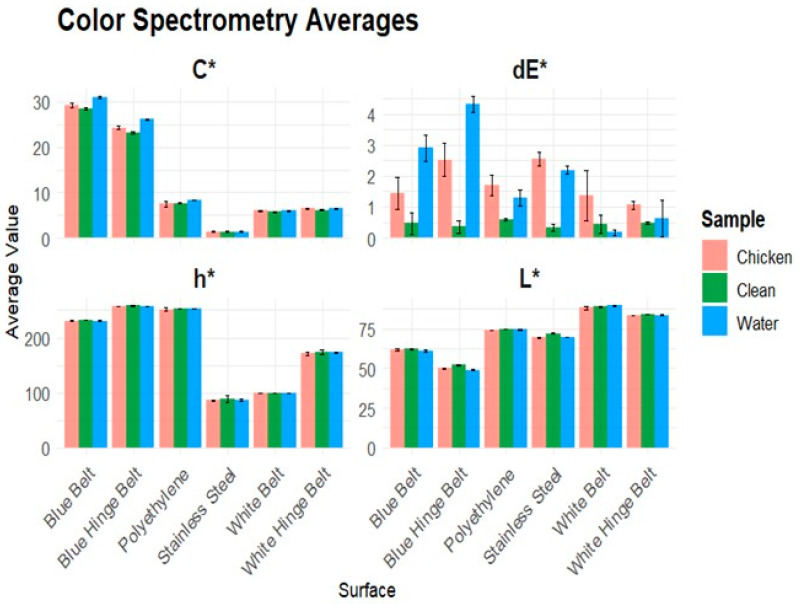
Display of the average and standard error of 30 color spectrometer measurements for six different surface types on the CIE-L*C*h* scale with dE*. Standard error was calculated using ten readings at three locations on each surface type.

Figure 2 visually shows the results of the CIE-L*C*h (lightness, chroma, hue) readings, along with the calculated color difference dE*.

The color spectrometer results in Figure 2 show that each surface creates different baseline effects for each sample. The dE* values were higher for water and chicken samples than for the clean baseline on each surface. The blue belt and blue-hinged belt created the most significant difference when comparing the clean sample to chicken and water for both chroma and dE*. The stainless steel had the most significant difference in hue, with the clean surface exhibiting a higher hue measurement compared to the chicken and water. The blue-hinge belt and stainless steel had the most significant decrease in lightness when chicken or water was added to the tested clean surface. The average color measurement values for each sample were surface-dependent, which resulted in distinguishable changes in color. The bio-fluorometer had significant results in the 280–340 nm channel F when chicken contamination was present due to tryptophan. The stainless steel surface had very little background fluorescence due to the material of the belt having no fluorescent compounds. This resulted in a significant amount of fluorescent intensity when chicken contamination was present. The pilot plant plastic belt had a significantly greater fluorescent intensity when chicken contamination was present in the same channel as the stainless steel. This proves that the tryptophan was the cause of channel F to have a high fluorescent intensity. The background fluorescence was due to fluorescent materials the belt was made of. Even when chicken contamination was present each channel except for 280–340 nm, it resulted in similar fluorescent intensities. The ability for specific residue detection is possible due to chicken contamination only causing a significant fluorescence intensity in the 280–340 nm channel. When inoculated with two sanitizing agents, PAA and QUAT, we obtained very similar results to the clean and water surface. This is caused by the sanitizing agents have no fluorescent chemicals in the solution allowing for only chicken contamination to be the only factor influencing the fluorescence intensity results.

We used the total color differences to calculate significant differences among samples tested on each of the six food-grade surfaces (Table 1). The total color difference (dE*) combines the lightness, chroma, and hue. When water is present on the blue belt and blue-hinge belt, the dE* mean values are higher than the clean and dry belts when chicken contamination is present. The clean and dry belt is the standardized sample, meaning that it should have a very small change in the dE* and only be affected by the consistency of the belt. The presence of water intensifies the color resulting in a change in the chroma due to the intensity of the saturation changing causing the highest dE* value. When chicken contamination is present, the change in color saturation decreases, causing a lower dE* value. The calculated dE* samples of clean and water samples were very similar to each other on the white belt and white hinge surface due to the water having very small effects on the color. The standard deviation on the blue belt, white belt, and white-hinge belt were also very similar to the calculated value of dE*. This was caused by the variance in the samples recorded to be very small, resulting in a small dE* along with small standard deviations within the readings of the instrument. The polyurethane plastic beltings were tested and showed results similar to those of stainless steel and polyethylene surfaces, with significant differences in fluorescent intensities between clean and chicken-contaminated samples. The poultry pilot plant belt is made of polyurethane to simulate the use of surfaces commonly used in poultry processing plants. The fluorometer is surface specific for each material the surface is made of, such as stainless steel, polyurethane, or polyethylene.

Each pairwise Tukey HSD test compares the average dE* value of each sample clean, water, or chicken (1:50 dilution). Significant differences were found for each sample within the stainless steel, blue belt, blue hinge, and polyethylene surface for dE* values. For both white surfaces, the color spectrometer could only differentiate chicken contamination on the surface.

The limit of detection (LOD) for humans to detect poultry contamination was found to be at a 1:50 dilution. The color spectrometer had positive results at distinguishing which surface contained poultry contamination at the human’s LOD by using the total color difference. The LOD of the color spectrometer was dependent on the surface, with most surfaces being between a 1:50–1:100 dilution.

### 3.2. Bio-Fluorometer

The handheld bio-fluorometer showed promising results in being able to detect chicken contamination that was also below that of a human’s sensory capabilities. The bio-fluorometer was tested using the same methods as testing color spectrometry. Two additional sanitizing compounds, in addition to water, were tested on each surface, including chicken soil. Each clean, water, cleaning compound, and chicken fluorescence intensity was compared within each food grade surface.

**Figure 3:**
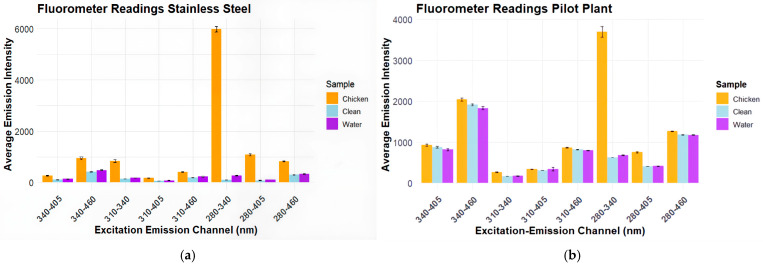
Average emission intensity of each excitation emission channel with standard error of 50 readings of chicken, clean, and water samples on each surface. (**a**) Fluorescence results on stainless steel; (**b**) fluorescence results on plastic pilot plant belt.

Figure 3 shows the fluorescent intensities for each excitation emission channel used by the bio-fluorometer on stainless steel.

The results in Figure 3a show a very high intensity of almost 6000 arbitrary units (A.U.) for chicken detection in the excitation emission channel of 280 nm–340 nm. The clean surface and water on stainless steel had very low emission intensities, never exceeding 1000 A.U. Across each excitation channel, only the channel 280 nm–340 nm had significant increases in fluorescence intensity. Figure 3b shows similar results, but with higher averages across each channel. In Figure 3b, the excitation emission channel 280 nm–340 nm still had significant differences between the chicken as compared to clean and water.

Based on the results of testing on stainless steel and the pilot plant belt, the excitation-emission channel 280 nm–340 nm was selected for the remainder of the bio-fluorometer testing.

The results of the Tukey test in Table 2 show each chicken dilution on stainless steel was significantly different than all clean samples. Clean, water, PAA, and QUAT all had much lower fluorescent intensities than model chicken soil for the emission excitation channel 280 nm–340 nm. For dilutions 1:40 and 1:50, the fluorescence means were found to be not significantly different from one another. The polyethylene results in Table 2 show higher overall fluorescence intensities than the stainless steel surface. Each chicken dilution except 1:10 had significantly different fluorescence means than the clean surfaces or surfaces with cleaning agents. The 1:25 solution was found to not be significantly different from either the 1:40 or 1:50 dilution.

The bio-fluorometer was able to detect poultry contamination at the human’s LOD on each of the surfaces. Due to background fluorescence on the polyurethane and polyethylene surfaces, the LOD for the bio-fluorometer was 1:50 at a minimum, with some of the plastic surfaces reaching to a 1:100 dilution LOD. The stainless steel surface was able to detect chicken contamination at a 1:100 dilution reliably making the LOD on stainless steel surfaces potentially greater than 1:100.

### 3.3. Human Sensory

Human sensory testing was completed by the authors only, as a formal sensory panel was not necessary. The results of the sensory testing allowed us to understand the conditions in a poultry processing plant and at what dilution a human could detect contamination. At a 1:50 dilution or beyond chicken contamination was no longer detected as the sight, smell, and odor was like that of water.

The strongest fluorescence intensities were on the polyethylene surface due to the components of the material creating background fluorescence. The polyurethane created stronger signals than the stainless steel surfaces, but was still less than the polyethylene’s background fluorescence. For industrial use, the most important factor is the type of surface being tested and understanding the amount of background fluorescence the surface type emits. The surface type being used provides the largest amount of potential error due to background fluorescence making understanding which surface is being tested a necessity. To improve the results for industrial use, consider taking preliminary readings on each clean surface you would like to test, test the instrument for when contamination is present, and then use the model to create each surface-specific analysis of clean or contaminated surfaces. After the specific surface has been characterized, it is possible to determine whether a valid assessment of its cleanliness can be achieved..

## 4. Discussion

The alternative methods of color spectrometry and bio-fluorescence technology can potentially be used to detect very low levels of chicken contamination on food-contacting surfaces after cleaning. Chicken processing plants are dark, wet, and can potentially limit human sensory abilities [6]. In a laboratory setting, chicken soil was not detected using human sensory abilities at a dilution of 1:50 chicken to water. Incorporating the instrument helped improve verification that contamination from chicken has been removed after the cleaning process.

ATP bioluminescence is a rapid test being able to obtain quantitative microbial contamination results in less than 1 min at a relatively low cost [19]. Measured in relative light units (RLUs), ATP bioluminescence has limitations of being dependent on swab type, surface type, operator confidence, chemical interferences, and nonspecific, resulting in too many variables to have a universal pass-fail threshold [20]. ATP bioluminescence limitation of being nonspecific causes false-positive results for harmless residues or false-negative results for residues that contain little amounts of ATP [19]. The correlation of with standard plate counts is inconsistent and cannot replace microbial testing as a proof of sanitation [21]. Chemical sanitizers, cleaning agents, or surface materials can cause variability and unreliable results for bioluminescence testing [22]. Color and fluorescent spectrometry are both compared to ATP bioluminescence as being rapid results in plant testing ability, quantitative, and at a higher cost. Fluorescent spectrometry can be protein specific being able to detect specific amino acids such as tryptophan while having no interference from cleaning agents. Only surface-dependent fluorescent spectrometry has the potential for a pass–fail threshold using a learning model specific for each surface type.

Color spectrometry has commonly been used to evaluate changes in the color of chicken meat [23]. In this study, color spectrometry had statistically significantly better results for detecting chicken soil on multiple food-contacting surfaces. Using the color scale L*a*b* to obtain a full color range of lightness, the chromaticity of red–green (a*), and chromaticity of yellow–blue (b*) were used to calculate the chroma and hue [24]. Hue is the term to define the color, and chroma, the intensity of the color, was used to measure changes in myoglobin pigments, resulting in our selection of the color scale CIE L*C*h [25]. Chroma and dE* had the best results for detecting chicken contamination in our study. Chroma in color imaging was able to detect chicken residue based on the fat identification properties [26]. The ΔE* results indicate statistically significant differences among the mean values of measurements obtained from stainless steel, polyethylene, and each blue-colored food-contact surface.. Since dE* is the calculated total color difference, it takes into effect all changes of the color, making it best for detecting any differences that may be found on a surface [27]. Issues of color spectrometry being tested on surfaces are potential changes in the surface due to degradation, scratching, or chipping [28].

Previous research has used fluorescence technology to detect invisible contaminants to aid in monitoring the cleanliness of institutional kitchens, in conjunction with adenosine triphosphate (ATP) swabs [29]. Fluorescence technology is emerging as a key component in cleaning and sanitation monitoring systems, and its application in the poultry industry is particularly promising. The amino acids tryptophan, tyrosine, and phenylalanine are commonly found in poultry meat [30]. Among these three amino acids, tryptophan exhibits the most pronounced fluorescent properties [31]. During this study, the experimental results show that tryptophan has the highest fluorescent peak within 20 nm of 280 nm excitation [15]. Our results confirm this, with the bio-fluorometer exhibiting the highest peak for chicken detection in the excitation-emission channel (280 nm–340 nm). Fecal contamination on meat carcasses was detected using a deep learning fluorescence imaging system, which faced the challenges of false-positives or false-negatives due to the variety of background surfaces in processing plants [32]. High-density polyethylene was found to exhibit fluorescence with an emission peak at 350 nm [33]. Background fluorescence was more commonly observed on plastic surfaces, with higher fluorescent intensities. Deep learning models have achieved 98.8% accuracy in detecting food residues on surfaces and equipment in food processing facilities [34]. A deep learning model has been implemented in the bio-fluorometer to help distinguish between clean and dirty data points. A support vector machine (SVM) was selected to train and test using data collected with the bio-fluorometer to classify surfaces as clean or dirty. The support vector model was included in this research to distinguish when a surface was clean or contaminated with chicken soil. Only preliminary tests of the embedded classifier model were completed to determine if the model validation had positive results. Through the preliminary results, a positive relationship was found in determining when a surface was clean or contaminated; however, more research is needed to provide statistical evidence of these results. Further testing will be required to enhance model accuracy and testing capabilities.

## 5. Conclusions

This study demonstrates that both color and fluorescence spectrometry offer rapid, objective, and highly sensitive methods for detecting chicken residues on food-contacting surfaces in poultry-processing environments. These technologies surpass traditional human sensory evaluation and ATP swabbing in their ability to identify contamination at levels below the threshold of human detection, particularly on stainless steel and blue plastic surfaces. Through exploratory preliminary results, the integration of machine learning classifiers may further enhance detection accuracy, enabling reliable differentiation between clean and contaminated states. These findings support the adoption of spectrometric tools as part of routine hygiene verification protocols, providing a more robust and quantifiable approach to ensuring food safety. Future research should focus on expanding the application of these methods to detect biofilms and other persistent contaminants, as well as optimizing learning models for diverse processing surfaces. By advancing these technologies, the poultry industry can move toward more effective, data-driven sanitation practices that reduce the risk of foodborne illness and improve overall plant hygiene.

## Figures and Tables

**Table 1:** Color spectrometry dE* results for each surface type with each surface sample.

Setup ID	Surface Sample	dE* Mean ± (SD)
Stainless Steel	Clean	0.3 ± (0.1) ^a^
	Water	2.2 ± (0.1) ^b^
	Chicken	2.6 ± (0.2) ^c^
Blue Belt	Clean	0.5 ± (0.4) ^a^
	Water	2.9 ± (0.4) ^b^
	Chicken	1.4 ± (0.5) ^c^
Blue Hinge	Clean	0.4 ± (0.2) ^a^
	Water	4.3 ± (0.3) ^b^
	Chicken	2.5 ± (0.5) ^c^
Polyethylene	Clean	0.6 ± (0.03) ^a^
	Water	1.3 ± (0.3) ^b^
	Chicken	1.7 ± (0.3) ^c^
White Belt	Clean	0.4 ± (0.3) ^a^
	Water	0.2 ± (0.1) ^a^
	Chicken	1.4 ± (0.8) ^c^
White Hinge	Clean	0.5 ± (0.04) ^a^
	Water	0.6 ± (0.6) ^a^
	Chicken	1.1 ± (0.1) ^b^

Lowercase letters indicate a significant difference among the averages of each sample’s dE* (pairwise Tukey HSD test, *p* < 0.05).

**Table 2:** Fluorescence intensity means of each sample compared with pairwise Tukey HSD test within stainless steel and polyethylene surfaces. All means and standard deviations are in A.U. (arbitrary units).

Surface	Sample	Fluorescence Mean ± (SD)
Stainless Steel	Clean	226 ± (138) ^a^
	Water	481 ± (164) ^a^
	1:10	11,200 ± (945) ^b^
	1:25	9430 ± (736) ^c^
	1:40	6400 ± (1550) ^d^
	1:50	6280 ± (338) ^d^
	PAA	170 (30.3) ^a^
	QUAT	161 (28.5) ^a^
Polyethylene	Clean	4820 ± (150) ^a^
	Water	4820 ± (254) ^a^
	1:10	5250 ± (1050) ^a^
	1:25	9460 ± (938) ^bc^
	1:40	10,100 ± (1420) ^b^
	1:50	9170 ± (1130) ^c^
	PAA	4820 ± (259) ^a^
	QUAT	5210 ± (171) ^a^

Lowercase letters indicate a significant difference among the means of each sample’s fluorescent intensity (pairwise Tukey HSD test, *p* < 0.05).

## Data Availability

The raw data supporting the conclusions of this article will be made available by the authors on request.

## References

[1] Code of Federal Regulations (CFR) Title 9 Animals and Animal Products, Part 416.11. https://www.ecfr.gov/current/title-9/chapter-III/subchapter-E/part-416.

[2] Code of Federal Regulations (CFR) Title 9 Animals and Animal Products, Part 416.12. https://www.ecfr.gov/current/title-9/chapter-III/subchapter-E/part-416.

[3] Schmidt R.H. (1997). Basic Elements of Equipment Cleaning and Sanitizing in Food Processing and Handling Operations. University of Florida Cooperative Extension Service, Institute of Food and Agriculture Sciences, EDIS. https://ucfoodsafety.ucdavis.edu/sites/g/files/dgvnsk7366/files/inline-files/26501.pdf.

[4] Scallan Walter E.J., Cui Z., Tierney R., Griffin P.M., Hoekstra R.M., Payne D.C., Rose E.B., Devine C., Namwase A.S., Mirza S.A. (2025). Foodborne Illness Acquired in the United States-Major Pathogens, 2019. Emerg. Infect. Dis..

[5] Scharff R.L. (2020). Food Attribution and Economic Cost Estimates for Meat- and Poultry-Related Illnesses. J. Food Prot..

[6] Harmse J.L., Engelbrecht J.C., Bekker J.L. (2016). The Impact of Physical and Ergonomic Hazards on Poultry Abattoir Processing Workers: A Review. Int. J. Environ. Res. Public Health.

[7] Marriott N.G., Schilling M.W., Gravani R.B. (2018). Meat and Poultry Plant Sanitation. Principles of Food Sanitation.

[8] Bland R., Brown S.R.B., Waite-Cusic J., Kovacevic J. (2022). Probing antimicrobial resistance and sanitizer tolerance themes and their implications for the food industry through the *Listeria monocytogenes* lens. Compr. Rev. Food Sci. Food Saf..

[9] Andersen C.M., Rasmus B. (2003). Practical aspects of PARAFAC modeling of fluorescence excitation-emission data. J. Chemom..

[10] Sueker M., Stromsodt K., Gorji H.T., Vasefi F., Khan N., Schmit T., Varma R., Mackinnon N., Sokolov S., Akhbardeh A. (2021). Handheld Multispectral Fluorescence Imaging System to Detect and Disinfect Surface Contamination. Sensors.

[11] Hwang C., Mo C., Seo Y., Lim J., Baek I., Kim M.S. (2021). Development of Fluorescence Imaging Technique to Detect Fresh-Cut Food Organic Residue on Processing Equipment Surface. Appl. Sci..

[12] Hunterlab (2008). CIE-L*a*b* Color Scale.

[13] Zhang Y.M., Hopkins D.L., van de Ven R., LUO X. (2018). Characterization of pH decline and meat color development of beef carcasses during the early postmortem period in a Chinese beef cattle abattoir. J. Integr. Agric..

[14] Rebane O., Babichenko S., Bentahir M., Poryvkina L., Gala J.L. (2021). Non-contact real-time detection and monitoring of microbial contaminants on solid surfaces using medium-(BC-Sense) and short-distance (H2B-Spectral) range sensors. Prime Archives in Public Health.

[15] Liu Y., Xu J., Han L., Liu Q., Yang Y., Li Z., Lu Z., Zhang H., Guo T., Liu Q. (2020). Theoretical Research on Excited States: Ultraviolet and Fluorescence Spectra of Aromatic Amino Acids. Interdiscip. Sci..

[16] Koh H.Y., Yu I.J. (2015). Nutritional Analysis of Chicken Parts. https://www.e-jkfn.org/journal/view.html?spage=1028&volume=44&number=7.

[17] Rõõm R. (2024). Distinguishing Bacteria from Fluorometer Spectra Using Machine Learning. Master’s Thesis.

[18] R Core Team (2024). R: A Language and Environment for Statistical Computing.

[19] Bakke M. (2022). A Comprehensive Analysis of ATP Tests: Practical Use and Recent Progress in the Total Adenylate Test for the Effective Monitoring of Hygiene. J. Food Prot..

[20] Osimani A., Garofalo C., Clementi F., Tavoletti S., Aquilanti L. (2014). Bioluminescence ATP Monitoring for the Routine Assessment of Food Contact Surface Cleanliness in a University Canteen. Int. J. Environ. Res. Public Health.

[21] Aycicek H., Oguz U., Karci K. (2006). Comparison of results of ATP bioluminescence and traditional hygiene swabbing methods for the determination of surface cleanliness at a hospital kitchen. Int. J. Hyg. Environ. Health.

[22] MilliporeSigma Do Disinfectants and Sanitizers Interfere with ATP Testing in the Food Industry? Application Note. “The Effects of Eight Frequently Used Compounds of Disinfectants, at Both Low and High Working Concentrations, on Commercial ATP-Based Hygiene Monitoring Systems. https://www.sigmaaldrich.com/deepweb/assets/sigmaaldrich/marketing/global/documents/102/370/atp-testing-in-food-industry-an12922en-ms.pdf?srsltid=AfmBOorO0OvFmQi0cLhLpNCuW_oT88-fhNQeQSPt6KRqEqETxGgBS-aL.

[23] Qamar A., Mohyuddin S.G., Hamza A., Lartey K.A., Shi C.Q., Yang F., Lu Z., Yang J., Chen J.J. (2019). Physical and chemical factors affecting chicken meat color. Pak. J. Sci..

[24] Andrade M.A., Barbosa C.H., Souza V.G.L., Coelhoso I.M., Reboleira J., Bernardino S., Ganhão R., Mendes S., Fernando A.L., Vilarinho F. (2021). Novel Active Food Packaging Films Based on Whey Protein Incorporated with Seaweed Extract: Development, Characterization, and Application in Fresh Poultry Meat. Coatings.

[25] Warner R., Klinth J.W., Dikeman M., Devine C. (2014). Measurement of water holding capacity and color: Objective and subjective. Encyclopedia of Meat Sciences.

[26] Edwards K., Manley M., Hoffman L.C., Williams P.J. (2021). Non-Destructive Spectroscopic and Imaging Techniques for the Detection of Processed Meat Fraud. Foods.

[27] Tkacz K., Modzelewska-Kapituła M., Więk A., Nogalski Z. (2020). The Applicability of Total Color Difference ΔE for Determining the Blooming Time in Longissimus Lumborum and Semimembranosus Muscles from Holstein-Friesian Bulls at Different Ageing Times. Appl. Sci..

[28] Breheny C., Colbert D.M., Bezerra G., Geever J., Geever L.M. (2025). Evaluating the Chemical Resistance and Performance of Thermochromic Polymers for Food Packaging. Materials.

[29] Hu B., Woods L., Qin J., Chan D.E., Baek I., Kim M.S., Vasefi F., Husarik K., Tavakolian K., Zadeh H.K. Enhancing cleanliness monitoring in institutional kitchens: A novel UV fluorescence imaging technology complementing adenosine tri-phosphate (ATP) swab tests. Proceedings of the SPIE PC13060, Sensing for Agriculture and Food Quality and Safety XVI, PC130600I.

[30] He W., Li P., Wu G. (2021). Amino Acid Nutrition and Metabolism in Chickens. Adv. Exp. Med. Biol..

[31] Grigoryan K.R., Shilajyan H.A. (2017). Fluorescence 2D and 3D spectra analysis of tryptophan, tyrosine and phenylalanine. Proc. YSU B Chem. Biol. Sci..

[32] Gorji H.T., Shahabi S.M., Sharma A., Tande L.Q., Husarik K., Qin J., Chan D.E., Baek I., Kim M.S., MacKinnon N. (2022). Combining deep learning and fluorescence imaging to automatically identify fecal contamination on meat carcasses. Sci. Rep..

[33] Arenas-Vivo A., Beltrán F.R., Alcázar V., de La Orden M.U., Urreaga J.M. (2017). Fluorescence labeling of high density polyethylene for identification and separation of selected containers in plastics waste streams. Comparison of thermal and photochemical stability of different fluorescent tracers. Mater. Today Commun..

[34] Gorji H.T., Van Kessel J.A.S., Haley B.J., Husarik K., Sonnier J., Shahabi S.M., Zadeh H.K., Chan D.E., Qin J., Baek I. (2022). Deep learning and multiwavelength fluorescence imaging for cleanliness assessment and disinfection in Food Services. Front. Sens..

